# Contradictions and Opportunities: Reconciling Professional Identity Formation and Competency-Based Medical Education

**DOI:** 10.5334/pme.1027

**Published:** 2023-11-06

**Authors:** Robert Sternszus, Natasha Khursigara Slattery, Richard L. Cruess, Olle ten Cate, Stanley J. Hamstra, Yvonne Steinert

**Affiliations:** 1 Department of Pediatrics & Institute of Health Sciences Education, McGill University, Montreal, Quebec, CA; 2 Mid-West Intern Network, University of Limerick, Limerick, IE; 3 Department of Orthopedic Surgery & Institute of Health Sciences Education, McGill University, Montreal, Quebec, CA; 4 Utrecht Center for Research and Development of Health Professions Education, University Medical Center Utrecht and Utrecht University, NL; 5 Department of Surgery, University of Toronto, Toronto, Canada; 6 Sunnybrook Research Institute, Holland Bone and Joint Program, Toronto, Canada; 7 ACGME, Chicago, IL, US; 8 Department of Medical Education, Northwestern University Feinberg School of Medicine, Chicago, IL, US; 9 Department of Family Medicine & Institute of Health Sciences Education, McGill University, Montreal, Quebec, CA

## Abstract

The widespread adoption of Competency-Based Medical Education (CBME) has resulted in a more explicit focus on learners’ abilities to effectively demonstrate achievement of the competencies required for safe and unsupervised practice. While CBME implementation has yielded many benefits, by focusing explicitly on what learners are doing, curricula may be unintentionally overlooking who learners are becoming (i.e., the formation of their professional identities). Integrating professional identity formation (PIF) into curricula has the potential to positively influence professionalism, well-being, and inclusivity; however, issues related to the definition, assessment, and operationalization of PIF have made it difficult to embed this curricular imperative into CBME. This paper aims to outline a path towards the reconciliation of PIF and CBME to better support the development of physicians that are best suited to meet the needs of society.

To begin to reconcile CBME and PIF, this paper defines three contradictions that must and can be resolved, namely: (1) CBME attends to behavioral outcomes whereas PIF attends to developmental processes; (2) CBME emphasizes standardization whereas PIF emphasizes individualization; (3) CBME organizes assessment around observed competence whereas the assessment of PIF is inherently more holistic. Subsequently, the authors identify curricular opportunities to address these contradictions, such as incorporating process-based outcomes into curricula, recognizing the individualized and contextualized nature of competence, and incorporating guided self-assessment into coaching and mentorship programs. In addition, the authors highlight future research directions related to each contradiction with the goal of reconciling ‘doing’ and ‘being’ in medical education.

Professional Identity Formation (PIF) is a burgeoning area of scholarly interest, as demonstrated by a large number of recent publications in medical education [1]. However, despite widespread recognition of its importance, PIF has not been systematically integrated into the structure of undergraduate and postgraduate medical education curricula [1,2]. This paradox is in no small part due to the challenge of ‘fitting’ PIF into the predominant curricular paradigm which emphasizes observable outcomes and competencies [3,4].

While outcomes-based movements in medical education have been described since the 1970s [5], they rose to prominence in the late 20^th^ century [6]. Reports such as ‘To Err is Human’ from the Institute of Medicine [7], as well as several high-profile cases in which the medical profession failed to meet its duties to society [8], brought forward many calls to action, both in healthcare and in medical education. These calls highlighted that variability in the quality of residency education needed to be addressed. This led to the emergence of outcomes frameworks (e.g., CanMEDS Roles, ACGME Milestones) [9,10] that explicitly defined the knowledge, attitudes, skills, and roles required to be a physician [11]. These frameworks became the pillars upon which learning objectives and educational experiences were created. In parallel, many programs paid attention to the need to explicitly teach and assess professionalism at all levels of medical education [1,2,3,4,5,6,7,8,9,10,11,12,13,14,15,16]. This ultimately led to the inclusion of professionalism into most competency frameworks [17].

Over the last decades, the outcomes movement has continued to flourish with the emergence of Competency-Based Medical Education (CBME), which further entrenched competencies as the primary focus of curricula [18,19]. CBME is an approach to preparing physicians to best meet the needs of society that is fundamentally oriented to the observation of abilities which are organized around the previously described outcomes frameworks [18]. It represents a shift from emphasizing only the successful completion of a number of discrete clinical experiences or surgical cases to making the explicit achievement of the competencies required for safe unsupervised practice, the primary objective [18,19].

There have been many benefits that have resulted from the move to CBME. Notably, it has enabled the medical profession to better meet its societal obligation by attempting to ensure the overall competence of those entering medical practice in a more consistent and systematic fashion [11,20,21,22]. With regards to professionalism, it has allowed for a clearer description of the values currently held by the profession. It has also helped to articulate attitudes and behaviors that are essential to upholding the commitment physicians must have to patients, society, the profession, and themselves [14,15,16,20,21,22,23].

However, as curricula increasingly honed in on direct observation and observable competencies, a fundamental Tenet in CBME, those studying professionalism began to emphasize professional identities and professional identity formation rather than the explicit teaching and assessing of professionalism and professional behaviors alone [24,25]. Professional identity is a representation of self whereby the characteristics, values, and norms of the medical profession are internalized, negotiated, and/or adapted, resulting in thinking, acting, and feeling like a physician [25,26]. It is reflected both in how others perceive the learner and how the learner perceives themselves, and it is heavily informed by one’s personal identities [25,26]. PIF is the developmental process whereby this professional identity emerges over time, through socialization of an individual into a community of practice [4,25,26,27].

Supporting the process of PIF has many potential benefits for learners and patients. In addition to helping learners recognize and integrate the professional roles, norms and values required for safe and ethical practice, supporting PIF can foster well-being by helping learners develop greater awareness of their values and ground their work in meaning [28]. Supporting PIF also has the potential to create more inclusive and accepting learning environments by acknowledging and addressing the potential misalignment that may occur between aspects of one’s personal identities and certain norms of the profession [29].

Developing competence and supporting PIF both seem to have great importance to medical education. However, concern has been raised that as CBME has focused curricula on what learners ‘do’, who learners are and who they are becoming may have been unintentionally overlooked [3,4,22,30,31,32,33]. The challenge of emphasizing both ‘doing’ and ‘being,’ that is encapsulated by the discourses of CBME and PIF, has been described in the literature; however, few solutions have been proposed [4,25,31,32,33]. In this paper, we argue that CBME and PIF are not only compatible, they can also both be enhanced by reconciling one with the other.

## Defining Contradictions and Opportunities

To address how professional identity formation can be meaningfully and explicitly integrated into competency-based curricula, we must first explore why it has been so difficult to reconcile PIF and CBME. We will focus on 3 important contradictions drawn from our collective experience and from the CBME and PIF literature. While we acknowledge that our analysis does not reflect all possible challenges, our aim is to open a conversation that can inform future scholarship and curricular innovation in this important area. The contradictions are as follows:

1. CBME attends to behavioral outcomes whereas PIF attends to developmental processes.
2. CBME emphasizes standardization, whereas PIF emphasizes individualization.
3. CBME organizes assessment around observed competence whereas the assessment of PIF is inherently more holistic.

## Behavioral Outcomes vs. Developmental Processes

### Defining the contradiction

CBME is largely rooted in behaviorist traditions [34,35]. Notably, it is focused on outcomes, defined as a series of observable competencies that are achieved in stages. Further, the workplace is promoted as the primary environment to produce learning of these competencies through direct observation and feedback. Ultimately, learning in CBME is viewed as the observable demonstration of the achievement of the stated competencies or outcomes that make up the curriculum. In this paradigm, the processes by which one achieves competence appear to be of little importance if competence is demonstrated [18,19,20,21].

In contrast, professional identity formation is rooted in constructivism and developmental psychology. The influence of developmental theories, such as those proposed by authors like Erikson and Kegan, ground PIF in the process of how learners move towards increasingly complex understandings of themselves as people and health professionals [4,36,37]. There is no singular desired identity, but rather, there are diverse and unique identities in which certain core conceptions of competence and professionalism are shared. Therefore, the emphasis is not placed solely on the outcome, but also on the process of constructing one’s identity through developmental processes that are unique to each individual learner [25,26]. Given its focus on process, it is not obvious how PIF can be meaningfully reflected in CBME.

### Potential opportunities

Even though PIF emphasizes process over outcome, it might be helpful to consider what outcomes we are aiming for when suggesting that educational programs must explicitly support PIF. Turning to the literature, most curricular or faculty interventions in support of PIF invoke guided reflections, engaging in difficult conversations, storytelling, mentorship, and creative expression through the medical humanities [1,2,38,39,40,41]. Each intervention aims to actively engage the learner in various ways (e.g., discussing, writing, drawing), as they construct and deconstruct their evolving professional identities. In essence, it appears that educational approaches in support of PIF are based on the idea that if learners engage in the process, they will have a greater influence in defining their evolving professional identities.

With this in mind, we propose that a key desired outcome, when it comes to supporting PIF in education, is for learners to be able to engage in their own identity development, continuously and actively, in a way that supports the acquisition of an authentic, integrated, and functional sense of professional self. In adopting this process-based outcome as a primary goal of medical education, one can then begin to articulate required competencies for engagement. Examples could include but are not limited to the learner being able to: (1) describe the various influences on their professional socialization process; (2) identify their values and those of the profession; (3) recognize instances in which personal values and perceived professional norms/values are in conflict. These competencies can then begin to serve as a basis for both curricular design and assessment of PIF in better alignment with CBME principles. Allowing for the inclusion of process-based outcomes in CBME, such as the ones described above, may allow for a greater individualization of curricula; it may also open the door to explicitly including PIF within outcomes and competency frameworks.

Another potential opportunity would be to explore how CBME can better align with the constructivist and developmental approaches that underpin PIF. In fact, the notion of developmental ‘stages’ (Royal College of Physicians and Surgeons of Canada) [10] or ‘levels’ of milestone achievement (Accreditation Council for Graduate Medical Education) [9] are already embedded in many CBME frameworks; however, their incorporation is limited to the evolution of competencies over time. Invoking a broader definition of these ‘stages’ or ‘levels’ to incorporate notions of personal and professional identity development alongside the progressive attainment of competence seems worthwhile. For example, an emphasis can be placed in early stages of training on a learner’s ability to set goals, plan, and reflect, whereas in later stages of training, competency frameworks can emphasize the ability to embrace complexity and hold multiple viewpoints and ideologies simultaneously (derived from Kegan’s stages of development) [36].

### Future directions for research & scholarship

We hypothesize that a focus on process outcomes, such as learner engagement in PIF, and expanding conceptions of stages or levels of training to include notions of personal and professional development, represent potential bridges between PIF and CBME. However, many questions remain. For example, how does increasing engagement in one’s professional identity formation impact the experience of being and becoming a physician? Are there ways to best promote and support learner engagement? What abilities or competencies are needed to effectively engage in the socialization process? What process outcomes, other than engagement, could be considered when supporting PIF through education? How does PIF during training integrate with one’s continuing PIF when in practice? In addition, while there is some published work that defines developmental stages related to PIF in pre-medical and undergraduate medical students [42], how can these developmental stages be defined across the spectrum of medical education?

## Standardization vs. Individualization

### Defining the contradiction

The primary goal of CBME is to better meet the needs of patients and be accountable to society. CBME underscores a deep investment in ensuring achievement of standardized outcomes as a way of guaranteeing, to the profession and to the public, that physicians have the required level of competence to provide safe care [18,19,20,21]. This standardization, which serves as way of codifying professional norms and values, is without a doubt essential.

Similarly, the literature on PIF in medical education espouses the goal of supporting the development of physicians with professional identities that reflect societal needs [26]. However, PIF more deliberately and explicitly emphasizes the role of individuality in this process [31]. It is believed that as one joins a community of practice, often through the process of socialization, personal identities will shape how learners relate to the various norms of the community which are, in turn, shaped and codified by professional standards. Variably, learners will accept certain norms with ease, some with more difficulty, and some following negotiation and adaptation; and some norms will be rejected [26,29]. When examining the norms and values of the profession, there is more that unites than divides. For example, there is little, if any, discussion around the notions that physicians should aim to do no harm or that they should conduct themselves with honesty. However, there can be substantially more tension around what is considered ‘professional’ when it comes to dress codes, working hours, variations in technique or stye, altruism, leaves of absence, or self-care [43,44,45,46]. A focus on PIF has the potential to nurture individuality and diversity by valuing reflection on the ways in which individual learners, who bring their unique personal experiences and backgrounds, may struggle differently with the stated norms [47,48,49].

Multiple studies suggest that promoting a diverse healthcare workforce is of tremendous benefit to society. Diverse workforces improve patient satisfaction, trust, access to, and utilization of services by minoritized patients as well as the breadth and scope of research and innovation [47,48,50,51]. The above have important implications for health policies and addressing disparities and inequities in health outcomes [52,53]. The need to include diversity and support individuality in the workforce is vital and must be better balanced with the current emphasis on standardization if curricula are to produce physicians who can fully meet complex societal needs.

### Potential opportunities

A discussion of how to move forward must emphasize individualization explicitly while still acknowledging the vital role of standardization. Competency frameworks have traditionally tended to conceptualize competence as the accumulation of various competencies which can be defined uniformly [54,55]. However, the degree to which this is reflective of reality is questionable. Does competence really look the same across various contexts (e.g., healthcare systems, hospitals, residency programs, countries)? Is competency attainment uniformly expressed across individuals? These critically important questions are beginning to garner more attention in the medical education literature with increasing recognition that competence should be seen as a construct that has contextualized and individualized elements [56]. In a recent study looking at competence when a learner changes contexts, it was found that learners needed to understand the new environment, develop a sense of belonging and legitimacy in the environment (i.e., become accepted within the new community of practice), and then re-constitute their competence in relation to the new setting [57]. This contextualized view of competence, which explicitly integrates PIF by acknowledging the individualized notions of socialization and belonging to a community of practice, represents a more nuanced understanding of competence that may help bridge CBME and PIF.

The results of the above-mentioned study imply that supporting contextualized competence requires curricula to explicitly attend to the process of socialization. Doing so requires a shift from an isolated focus on discrete learning experiences (i.e., multiple rotations in multiple contexts with multiple supervisors) to also considering experiences that foster longitudinal relationships and allow time and space for reflection on belonging [58]. Examples could include the incorporation of longitudinal mentorship groups focused on the experience of integrating into the community of practice, or periodic, guided reflections with a mentor on one’s professional identity development and experiences, both positive and negative, that have been the most influential.

The above examples of curricular interventions could easily be integrated into local programs, and in some cases, already have been implemented successfully [1,38,39,40,41,59,60,61]. However, contextualized and individualized notions of competence (i.e., competence as a multi-layered concept) [62] must occupy an explicit space within the structure of CBME, alongside roles and domains, if activities designed to support individuality and PIF are to become mainstream.

### Future directions for research & scholarship

We hypothesize that adopting a PIF lens to re-imagine competence and competency frameworks has the potential to allow for the explicit acknowledgement of context, diversity, and individuality in the expression of competence. However, it is important to recognize that scholarship of PIF to date has not meaningfully included the experiences of learners underrepresented in medicine, or the role of power and the socio-political-cultural context in PIF [63,64]. Additional studies are needed if PIF is to foster individuality and diversity within CBME frameworks. For example, a greater understanding of how one’s individual identities shape conceptualizations of competence and how learners who are under-represented in medicine experience socialization would be key areas for future scholarship.

## Assessment as Observed Competence vs Holistic Assessment

### Defining the contradiction

Assessment in CBME is based on the observation of learner competencies in authentic workplace environments by multiple assessors and using multiple methods [65,66]. Each assessment is paired with timely and specific formative feedback to promote growth in the learner. Summative decision-making results from a compilation of these assessments via a collective group process (i.e., assessment committee). The result of this group process is the charting of an overall representation of learner competence. In other words, multiple small pixels, each taken from different angles and then pieced together by a committee, make up a representation of the whole in most CBME assessment programs [66]. While there have been many advantages to adopting CBME assessment models, a recurrent criticism is that they may be overly reductionist in their implementation and blind the assessment process to certain elements of competence and to who learners are becoming [3,4,22,30,31,32,33]. Recent discussions of prospective entrustment in CBME have begun to address components of competence that are not easily observed in discrete encounters (e.g., humility) [67,68]. However, the individualized and personalized nature of who and how learners become (i.e., PIF) is still not fully captured.

While assessment in CBME aspires to be holistic, PIF lends itself more naturally towards holistic assessment [69]. While the literature on assessment in PIF is scant, self-assessments and self-reflection appear to be the most oft used approaches [70,71,72,73]. Many challenges to the inclusion of PIF into programs of assessment have also been described. Examples include the lack of a standardized outcome (i.e., singular desired identity) and the difficulty of assessing internal processes such as how learners think and feel (i.e., because professional identity is defined as ‘thinking, acting, and feeling like a physician’) [73]. As such, a systematic approach to assessment in PIF has yet to materialize [74].

It appears that CBME curricula may be well-served by building more holistic forms of assessment into their frameworks. PIF holds such a view of assessment but practical and easily operationalized assessments of PIF have been difficult to develop [74]. Exploring the challenge of how holistic and discrete approaches to assessment can be better integrated appears critical to both CBME and PIF.

### Potential opportunities

It may be helpful to return to our definition of professional identity which emphasizes both how one sees oneself and how one is perceived by others. Focusing assessment on behaviors and performance observed by supervisors and other healthcare professionals, a cornerstone of assessment in CBME, can provide a reasonable surrogate of how others perceive the learner. This is especially true when narrative comments are meaningfully included in the summative group decision-making processes of assessment committees (not always the case) [66]. Therefore, placing additional emphasis on how learners see themselves, relative to how they are viewed by others, may be what is needed to better integrate PIF into the CBME assessment system and make assessment in CBME more holistic.

One potential opportunity that should be explored is the role and mandate of academic advisors or coaches. While a standardized definition of these roles is lacking, advisors or coaches are often faculty members who review and discuss assessment data with residents on a longitudinal basis in CBME programs. When adopted in this way, the goal is to generate a summary of performance including specific areas for improvement [75]. We believe there is an opportunity to explicitly include specific, guided self-assessment on how the learner sees themselves, beyond just notions of competence, in these relationships. In this way, the self-assessment will be guided by frequent oversight and feedback. For example, learners could reflect with their coaches or academic advisors, at regular intervals, on what the narrative comments in their assessments reveal about how they are perceived by others and the ways in which this does or does not align with how they view themselves. The evolution of these narrative comments and guided self-assessments could be charted by academic advisors or coaches to further inform the summative assessment processes of the assessment committees. In turn, this would provide a means for CBME programs to explicitly acknowledge that there is more to being a physician than ‘checking off’ all the competencies.

### Future directions for research & scholarship

We hypothesize that explicitly including guided self-assessments that move beyond competence could help operationalize how PIF can be included in a system of assessment and how assessment in CBME can be made more holistic. We have also suggested that a greater emphasis on narrative comments would be of importance. However, there are many questions that remain unanswered. For example, how can the literature on narrative assessments [76,77] be more meaningfully used by assessment committees in CBME? How can guided self-assessments be included in overall assessment without making it high-stakes and performative? What role should a coach or academic advisor, as we have imagined it, play in an assessment committee? How can multi-source feedback more reliably include multiple perspectives (e.g., patients, nurses, administrative assistants, peers) to best capture how others see learners? How can the alignment, or misalignment, between narrative comments and self-assessments contribute to learning and overall assessment of competence? Of all the contradictions outlined in this paper, this one appears to need the most ongoing work. Given today’s assessment culture, it also appears most vital to the meaningful reconciliation of PIF and CBME.

## Conclusions

In sum, the goal of shifting medical education towards CBME was to ensure that physicians are better able to meet societal needs. We propose that CBME will be enhanced and better positioned to meet this goal by explicitly incorporating PIF. Reconciling CBME and PIF has been a significant challenge due to several contradictions that need to be addressed. However, these paradigms are not incompatible and many opportunities to move forward exist. For example, articulating process-based outcomes for curricular initiatives focused on PIF to allow them to be encoded within competency frameworks, representing individualized and contextualized notions of competence in existing frameworks, emphasizing longitudinal mentorship and reflection, and incorporating guided self-assessment (i.e., how one sees themself) represent potential first steps. There is still much to be uncovered and we have proposed several potential scholarly questions to be examined. What is abundantly clear is that continuing this critical conversation is required if medical education is to be successful in fostering physicians’ individual identity formation while at the same time developing physicians that are best suited to meet the needs of the societies they serve.
